# Supplementary material for: TICI: a taxon-independent community index for eDNA-based ecological health assessment
Source: PeerJ. 2024 Feb 26;12:e16963. doi: 10.7717/peerj.16963 (PMC10903356; doi:10.7717/peerj.16963)
Supplement: File S1 [file peerj-12-16963-s001.docx]

**Supplementary file S1:** Site metadata table for 53 sampling locations included in the Summer 2020/2021 eDNA survey.

| **SiteID** | **SiteName** | **Authority** | **Latitude** | **Longitude** | **CollectionDate** | **Substrate** | **FiveYearMedianMCI** | **TICI** | **CV** |
| --- | --- | --- | --- | --- | --- | --- | --- | --- | --- |
| RC001 | Lower Rangitata | Environment Canterbury | -44.1875 | 171.5084 | 2021-02-09 | HB | NA | 109.68 | 0.017 |
| RC002 | Waituna Creek at White Pine Road | Environment Southland | -46.5202 | 168.5427 | 2021-03-23 | HB | 76 | 84.4 | 0.015 |
| RC003 | Oreti River at Branxholme | Environment Southland | -46.2955 | 168.2841 | 2021-03-23 | HB | NA | 96.24 | 0.01 |
| RC004 | Oreti River at McKellars Flat | Environment Southland | -45.3262 | 168.2085 | 2021-03-24 | HB | 114.3 | 112.03 | 0.006 |
| RC005 | Waikato River at Hamilton | Waikato Regional Council | -37.7938 | 175.2906 | 2021-01-25 | SB | NA | 77.74 | 0.025 |
| RC006 | Komakorau Stream | Waikato Regional Council | -37.6991 | 175.3299 | 2021-01-20 | SB | 76.9 | 77.92 | 0.022 |
| RC007 | Maketawa | Taranaki Regional Council | -39.2362 | 174.1844 | 2021-02-01 | HB | 135 | 124.77 | 0.004 |
| RC008 | Kaupkonui | Taranaki Regional Council | -39.5403 | 174.0826 | 2021-04-15 | HB | 93 | 97.56 | 0.008 |
| RC009 | Tangahoe | Taranaki Regional Council | -39.6291 | 174.3491 | 2021-04-15 | HB | 98 | 87.8 | 0.012 |
| RC010 | Nuhaka River | Hawke's Bay Regional Council | -39.0053 | 177.765 | 2021-03-31 | HB | 87.8 | 83.4 | 0.016 |
| RC011 | Waingongoro | Hawkes Bay Regional Council | -39.3822 | 176.3325 | 2021-03-19 | HB | 93.3 | 94.52 | 0.012 |
| RC012 | Kuripapango | Hawkes Bay Regional Council | -39.8078 | 176.9857 | 2021-03-21 | HB | 129.1 | 117.95 | 0.01 |
| RC013 | Papanui Stream | Hawkes Bay Regional Council | -39.8559 | 176.715 | 2021-03-26 | SB | 70 | 69.86 | 0.016 |
| RC014 | Fernhill | Hawkes Bay Regional Council | -39.588 | 176.77 | 2021-03-21 | HB | 100 | 96.25 | 0.028 |
| RC015 | Redjacks Creek | West Coast Regional Council | -42.438 | 171.4782 | 2021-03-01 | HB | 146 | 121.33 | 0.009 |
| RC016 | Okutua Stream | West Coast Regional Council | -43.2006 | 170.2664 | 2021-02-21 | HB | 122 | 122.06 | 0.013 |
| RC017 | Haast River | West Coast Regional Council | -43.9378 | 169.2958 | 2021-02-19 | HB | 119 | 126.84 | 0.008 |
| RC018 | Poormans Seaview | Nelson City Council | -41.303 | 173.226 | 2021-02-03 | HB | 84.5 | 92.99 | 0.01 |
| RC019 | Poormans Barnicoat | Nelson City Council | -41.317 | 173.25 | 2021-02-04 | HB | 115 | 96.97 | 0.011 |
| RC020 | Maitai Groom | Nelson City Council | -41.287 | 173.328 | 2021-02-04 | HB | 97 | 95.25 | 0.008 |
| RC021 | Wakapuaka at Hira | Nelson City Council | -41.214 | 173.399 | 2021-03-18 | HB | 106 | 105.17 | 0.007 |
| RC022 | Roding at Caretaker | Nelson City Council | -41.357 | 173.26 | 2021-04-09 | HB | NA | 118.63 | 0.008 |
| RC023 | Whangamoa Kokorua | Nelson City Council | -41.129 | 173.541 | 2021-02-23 | HB | 113.5 | 101.5 | 0.011 |
| RC024 | Waikawa at North Manakau Road | Horizons Regional Council | -40.707 | 175.2345 | 2021-03-10 | HB | 129 | 111.01 | 0.011 |
| RC025 | Tamaki at Reserve | Horizons Regional Council | -40.1201 | 176.0311 | 2021-02-24 | HB | 130 | 126.24 | 0.007 |
| RC026 | Turitea at Massey Farm Bridge | Horizons Regional Council | -40.3853 | 175.6079 | 2021-03-17 | HB | 94 | 83.07 | 0.009 |
| RC027 | Waiarohia | Northland Regional Council | -35.7443 | 174.2932 | 2021-02-02 | HB | 75.4 | 79.16 | 0.014 |
| RC028 | Tangowahine | Northland Regional Council | -35.8443 | 173.9359 | 2021-02-02 | HB | NA | 71.74 | 0.018 |
| RC029 | Waipoua | Northland Regional Council | -35.6525 | 173.5704 | 2021-01-29 | HB | 130.8 | 109.8 | 0.008 |
| RC030 | Ruakaka | Northland Regional Council | -35.8694 | 174.4026 | 2021-01-27 | SB | 77.5 | 71.01 | 0.015 |
| RC031 | Tapapa | Northland Regional Council | -35.193 | 173.4807 | 2021-01-28 | HB | 124.2 | 112.74 | 0.012 |
| RC032 | Are Are Creek | Marlborough District Council | -41.4674 | 173.8264 | 2021-02-24 | HB | 95.5 | 91.45 | 0.011 |
| RC033 | Lower Rakaia | Environment Canterbury | -43.8998 | 172.2071 | 2021-02-02 | HB | NA | 115.41 | 0.014 |
| RC034 | Lower Ashburton/Hakatere | Environment Canterbury | -44.0525 | 171.8045 | 2021-02-05 | HB | NA | 101.24 | 0.015 |
| RC035 | Avondale Stream | Auckland Council | -36.9236 | 174.6908 | 2021-03-17 | SB | 67.7 | 75.5 | 0.01 |
| RC036 | Piha Stream | Auckland Council | -36.9534 | 174.4756 | 2021-03-10 | HB | NA | 90.82 | 0.017 |
| RC037 | Ngakoroa Stream | Auckland Council | -37.1955 | 174.9736 | 2021-03-09 | SB | 75.5 | 77.6 | 0.015 |
| RC038 | Karaponga Stream | Bay of Plenty Regional Council | -38.0096 | 176.708 | 2021-04-30 | SB | 120 | 114.24 | 0.02 |
| RC039 | Mimiha Stream | Bay of Plenty Regional Council | -37.8672 | 176.6946 | 2021-04-23 | SB | 121 | 106.89 | 0.017 |
| RC040 | Landslip Creek | Environment Canterbury | -42.6921 | 172.0453 | 2021-03-31 | HB | NA | 113.24 | 0.008 |
| RC042 | Blue Duck Creek | Environment Canterbury | -42.2792 | 173.7669 | 2021-03-30 | HB | 113.6 | 99.53 | 0.008 |
| RC043 | Kauru River | Otago Regional Council | -45.1091 | 170.7418 | 2021-04-22 | HB | 116 | 108.32 | 0.007 |
| RC044 | Dunstan Creek | Otago Regional Council | -44.9395 | 169.7652 | 2021-05-03 | HB | 119 | 109.96 | 0.006 |
| RC045 | Clydevale | Otago Regional Council | -46.1014 | 169.5327 | 2021-05-04 | HB | NA | 90.7 | 0.019 |
| RC046 | Waipoua River | Greater Wellington Regional Council | -40.9536 | 175.673 | 2021-04-07 | HB | 102.5 | 90.31 | 0.012 |
| RC047 | Kaiwharawhara Stream | Greater Wellington Regional Council | -41.2599 | 174.7886 | 2021-05-07 | HB | 92.4 | 98.8 | 0.008 |
| RC048 | Whareroa Stream | Greater Wellington Regional Council | -40.9558 | 174.9702 | 2021-04-16 | SB | NA | 83.08 | 0.009 |
| RC049 | Wainui Stream | Greater Wellington Regional Council | -40.974 | 174.9616 | 2021-04-13 | HB | NA | 87.84 | 0.006 |
| RC050 | Ohutu Stream | Bay of Plenty Regional Council | -38.3908 | 176.8318 | 2021-05-10 | HB | NA | 118.82 | 0.007 |
| RC051 | Whanarua Stream | Bay of Plenty Regional Council | -37.6846 | 177.7887 | 2021-05-11 | HB | 128 | 120.48 | 0.016 |
| RC052 | Manganui Small | Waikato Regional Council | -38.5172 | 174.7105 | 2020-12-14 | HB | 132.4 | 126.82 | 0.011 |
| RC053 | Manganui Big | Waikato Regional Council | -38.5172 | 174.7105 | 2020-12-14 | HB | 141.2 | 124.73 | 0.012 |
| RC054 | Manganui SOE | Waikato Regional Council | -38.5937 | 174.6835 | 2020-12-15 | HB | 106.1 | 105.65 | 0.009 |
