# Supplementary material for: TICI: a taxon-independent community index for eDNA-based ecological health assessment
Source: PeerJ. 2024 Feb 26;12:e16963. doi: 10.7717/peerj.16963 (PMC10903356; doi:10.7717/peerj.16963)
Supplement: File S2 [file peerj-12-16963-s002.docx]

**Supplementary file S2:** Optimization of TICI indicator sequence depth

We selected the number of possible TICI indicator sequences as 3,000, based on a trade-off between accuracy and precision. While the addition of more indicator sequences would have marginally reduced the within-site CV values further (Fig. S2.1a), the mean CV was sufficiently minimal at 0.0125, the correlation with existing MCI values was near its maximum (5-year median MCI; R^2^ = 0.85; Fig. S2.1b), and there was a near-perfect correlation between the TICI values from the TICI3K, TICI5K and the TICI10K (Fig. S2.2). The 3,000th most prevalent sequence occurred in 5% of samples (45/848), while the 10,000th most prevalent sequence occurred in only 1.5% of samples (12/848).


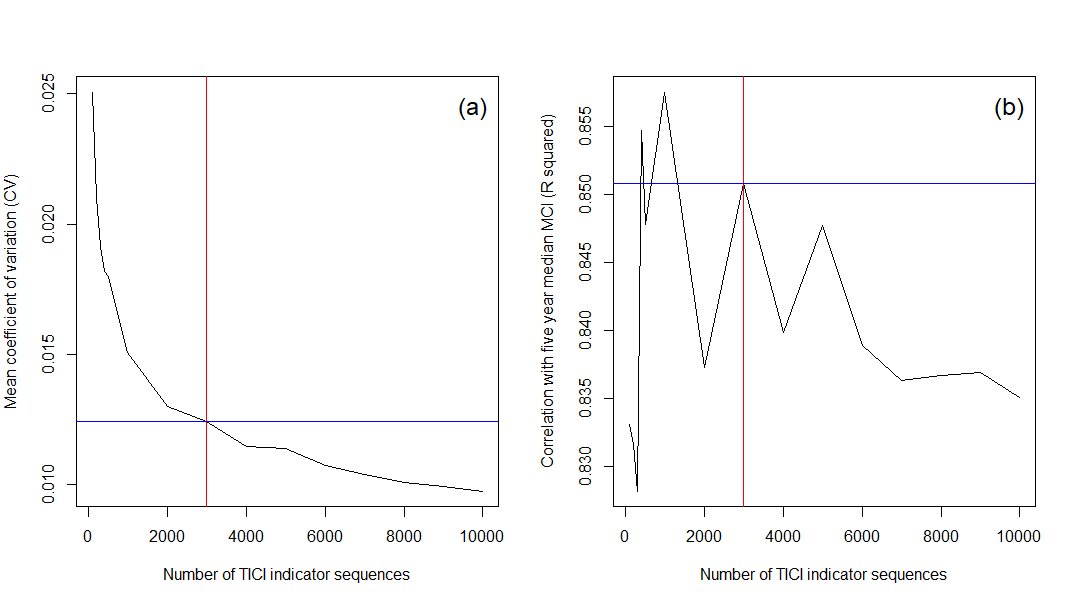


**Figure S2.1.** Diminishing effect of increased numbers of indicator sequences on variation in TICI values between site-replicates (a), and correlation of different TICI models with existing 5-year median MCI data (b). The TICI3K model (3,000 indicator sequences) was selected based on the diminishing inter-replicate CV, a strong correlation between the TICI3K values and the 5-year median MCI, and the near-perfect correlation of TICI values correlation matrix for TICI3K, TICI5K and TICI10K.


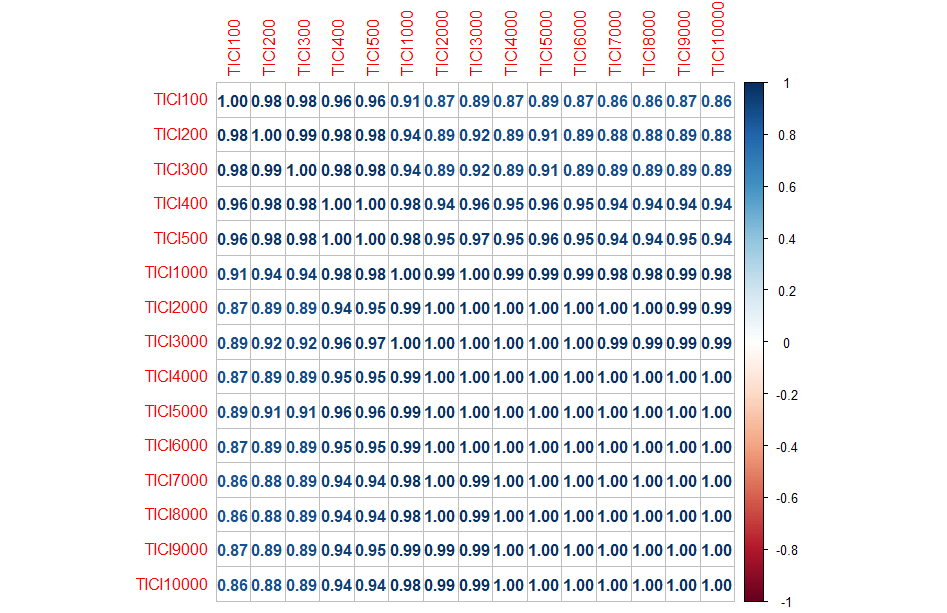


**Figure S2.2.** Correlation matrix of the TICI values of all 848 eDNA samples, across a gradient of indicator sequence numbers (i.e. using the *x* most commonly encountered sequences to construct a TICI*x*K value). Correlation matrix plotted using the ‘corrplot’ R package (Wei et al., 2017).
