# Supplementary material for: TICI: a taxon-independent community index for eDNA-based ecological health assessment
Source: PeerJ. 2024 Feb 26;12:e16963. doi: 10.7717/peerj.16963 (PMC10903356; doi:10.7717/peerj.16963)
Supplement: File S3 [file peerj-12-16963-s003.docx]

**Supplementary file S3:** Development of eDNA-based macroinvertebrate community index (eMCI) to approximate initial site quality rankings.

The macroinvertebrate community index (MCI) is currently the most widely used stream condition assessment method in New Zealand. There are ~220 taxa that contribute to the MCI index, mostly designated at genus level. These taxa have been assigned tolerance scores based on their observed tolerance to organic enrichment (MCI-hb: Stark 1985) and later the Chessman iterative learning process (MCI-sb; Stark and Maxted 1987). Invertebrate samples are generally collected from wadeable streams using a standardized kick-net protocol (NEMS 2022), preserved in isopropanol, and sent to a testing laboratory for identification. Indicator values are then averaged to produce a stream condition score for the site (Stark 1985; Stark and Maxted 1987).

The Chessman (2003) learning process requires sites to be initially ranked by quality using an existing condition metric; however, only 40 of the 53 sites in this study had multiple years of kick-net MCIs data available. It was therefore necessary to approximate the MCI using a the eDNA data in order to provide the initial site quality ranking. To achieve this, we used eDNA-derived taxon information in place of morphological IDs . For each sample, NCBI taxon IDs were converted to Stark and Maxted (2007) taxon categories using the get_lineage() function in the insect R package (Wilkinson et al., 2018). Individual tolerance scores derived from the eDNA data (i.e. eMCI scores) were then calculated for each sample by averaging the tolerance values and multiplying by 20 to produce the index value in the range of 0-200 as described in (Stark, 1985). Final site-eMCIs were calculated by averaging individual eMCIs for the 16 replicate eDNA samples within each site.

To test the ability of the eMCI to replicate the standard MCI for stream condition assessment, linear regression was applied to correlate the averaged eMCI scores against the historic kick-net MCI Scores. Historic scores were calculated as the 5-year median MCI for a subset of intensively monitored sites for which morphological MCI data were available (40 of the 53 sites). All regression analyses and plot generation were carried out in R (R Core Team 2021).

The site-averaged eMCI performed well against the standard kick-net sampling approach. The strong correlation between the 5-year median kicknet MCI and the eMCI  (R^2^ = 0.78) was surprising given the number of factors contributing to the residual variation between the morphological and molecular methods. For example, sources of variation to traditional MCI sampling include variation between sample collectors, as well as natural temporal variation to communities so that MCI values normally fluctuate. An extreme example of this is a difference of 40-units in MCI scores over subsequent years in the Maitai River at Groom Road in Nelson, South Island (LAWA ID ncc-00015; see [LAWA website](https://www.lawa.org.nz/explore-data/river-quality/)). A second factor acting to decouple the MCI and eMCI is that not all Stark MCI taxa are currently represented in the GenBank and BOLD databases. Thus, the eMCI values are not as robust as they might be if the reference database was in a more complete state. At the time of publication, only around ⅔ of the aquatic invert taxa listed in a recent paper have COI sequences deposited in GenBank and BOLD databases (Drinan et al., 2021). eDNA outputs also provide many more taxa than could reasonably be counted in a standard kicknet sample, including terrestrial taxa that may contribute to the eMCI but not the traditional kicknet method. For example, the Stark taxon list includes the phylum Oligochaeta (annelid worms, with an indicator score of 1.0), and it is very common for riverine eDNA samples to contain DNA from terrestrial earthworm species from the *Aporrectodea Eisenia* and *Lumbricus* genera. It is therefore more likely that an eMCI will include the oligochaete contribution of 1.0 than the standard kicknet-derived MCI. Despite these sources of variation, the relationship appeared sufficiently correlated and unbiased for SOE monitoring when averaging eMCI over 6 replicate samples.


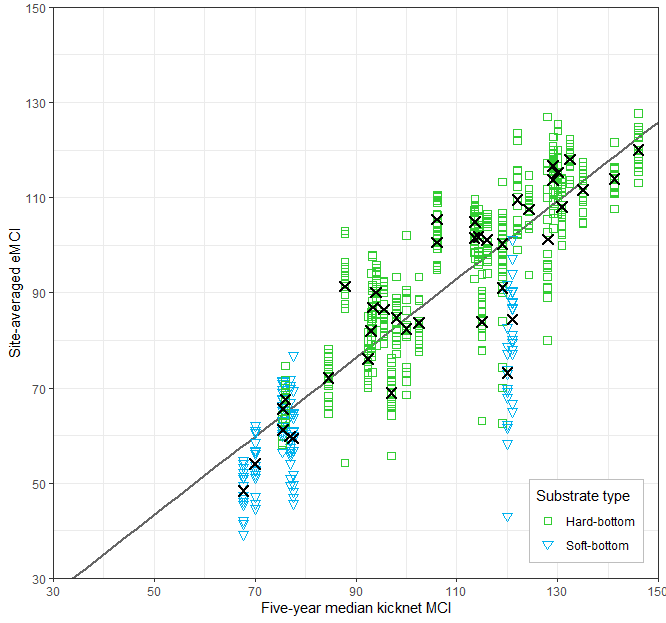


**Figure S3.1**. Validation of site-averaged eDNA-based MCI (eMCI) against five-year median kicknet MCI for 40 sites for which multiple years of data are available. Hard-bottomed sites are shown in green, and soft-bottomed sites in blue. The R^2^ value of 0.78 between 5-year median MCI and site-averaged eMCI (green points) indicates a moderate to strong correlation between methods, and demonstrates the utility of eMCI for hard-bottomed sites when scores are averaged over several sample replicates.
